# Integrating Multi-Temporal UAV Thermal Imaging and 3D Path Planning for Facade Thermal Defect Diagnosis in Old Residential Buildings

**DOI:** 10.3390/s26144385

**Published:** 2026-07-10

**Authors:** Senhong Cai, Xuetong Li, Zhonghua Gou

**Affiliations:** School of Urban Design, Wuhan University, Wuhan 430072, China; caisenhong@whu.edu.cn (S.C.); 2023282090031@whu.edu.cn (X.L.)

**Keywords:** UAV thermal imaging, multi-temporal analysis, 3D path planning, facade thermal defect diagnosis, thermal anomaly recognition, old residential buildings

## Abstract

Facade thermal defect diagnosis is a critical prerequisite for energy-efficiency retrofitting of old residential buildings. However, conventional infrared thermography is easily affected by environmental conditions and occupant behavior, making it difficult to distinguish persistent thermal defects from transient anomalies. To address this challenge, this study proposes an integrated diagnostic framework for old residential buildings in Wuhan, China, combining unmanned aerial vehicle (UAV) infrared thermography, multi-temporal data acquisition, 3D flight-path planning, thermal anomaly recognition, facade spatial mapping, and temporal screening. Field experiments were conducted to determine key acquisition parameters, including sensor preheating time, imaging distance, and acquisition timing. Thermal anomalies were identified through image-processing techniques and mapped onto facade representations derived from 3D models. Repeated observations across different times and days were then used to evaluate anomaly recurrence and spatial stability. The results show that preheating the sensor for at least 10 min, maintaining a UAV-to-facade distance of 8–10 m, and acquiring data around 17:00 provide more reliable thermal images. Multi-temporal screening effectively reduces false positives caused by temporary disturbances, while persistent anomalies associated with window–wall joints, floor slabs, wall surfaces, and moisture-related areas can be identified more robustly. The proposed framework provides a practical workflow for facade thermal defect diagnosis and retrofit-oriented decision support.

## 1. Introduction

The escalation of building energy consumption and the imperative to improve indoor thermal comfort have placed existing building envelopes under critical scrutiny, particularly in the context of urban renewal and carbon neutrality goals [1]. In China, national policies such as the “14th Five-Year Plan” for Building Energy Efficiency and local regulations like the Wuhan Green Building Management Measures mandate the energy retrofitting of old residential communities, emphasizing the enhancement of envelope thermal performance to reduce heat loss and improve occupant comfort [2,3]. However, for buildings in hot-summer and cold-winter (HSCW) zones, the long-term effects of solar radiation, thermal cycling, and moisture ingress often lead to material aging and construction degradation, resulting in localized thermal defects such as thermal bridges, air leakage, and insulation detachment [4,5]. These defects significantly undermine retrofit effectiveness. Therefore, accurately diagnosing facade thermal defects is a prerequisite for successful, sustainable renovation.

Infrared thermography (IRT), by detecting infrared radiation from object surfaces and converting it into temperature distribution images, provides a non-contact, rapid, and visual diagnostic tool for identifying thermal anomalies on building facades. Extensive research has applied IRT to various building envelope diagnostic scenarios. For thermal bridge and insulation-related defects, studies have demonstrated the effectiveness of IRT in identifying heat loss pathways and insulation detachment [6,7,8,9]. In the context of extreme climates, Aoul et al. [10] analyzed residential buildings under high-temperature conditions and found that thermal anomalies were more frequently observed in newly constructed buildings than in older ones, with defects concentrated at reinforced concrete-to-insulation interfaces and oversized repair points. For moisture-related issues, including wall dampness and air leakage, IRT has been successfully used to detect moisture infiltration paths and seal failures [11]. Martinez and Martinez [12] used passive IRT to assess timber structures in Spain, successfully identifying cracks, moisture anomalies, and surface-layer detachments, while Edis et al. [13] demonstrated that night-time surveys under stable temperature conditions are more effective for detecting moisture problems in ceramic-clad facades due to reduced solar interference. Additionally, IRT has shown promise in evaluating window air-tightness and finish layer conditions [14]. Fox et al. [15] conducted a large-scale comparative study on 122 residential buildings in England, concluding that walk-through surveys identify thermal defects more accurately than drive-by surveys, which are more susceptible to weather and viewing-angle variations. Compared with visual inspection, tapping methods, and localized destructive testing, IRT can present surface temperature field distributions over large areas and identify potential thermal defects through temperature differences, boundary morphology, and spatial locations [16,17].

Beyond qualitative defect identification, recent studies have also advanced the quantitative use of IRT for building energy performance assessment. Zhu et al. [18] demonstrated that IRT can be used to analyze the thermal performance of different wall materials and to compare energy efficiency across building envelope systems. Benhmidou et al. [19] combined IRT measurements with TRNSYS simulations to validate thermal conductivity data for existing buildings in northern Morocco, showing that thermographic data can support building energy modeling. Mohammad and Shea [20] further emphasized that under dynamic climatic conditions, envelope thermal performance assessment requires more detailed thermal behavior analysis rather than static judgments alone. Sadeghifam et al. [21] and Kisilewicz [22] showed that integrating IRT data with building energy simulation results provides a more complete understanding of envelope thermal performance and facilitates the translation of inspection results into retrofit decisions. The selection of acquisition timing has also been identified as a critical factor: de Freitas et al. [23] found that midday surveys under strong solar radiation are more effective for detecting facade plaster detachments, whereas Edis et al. [13] recommended night-time surveys for moisture detection due to more stable temperature differences between wet and dry areas.

However, a critical gap persists in the stability and reliability of these diagnoses under real-world conditions. A thermal image represents the surface thermal response under specific boundary conditions, not the defect itself. Solar radiation, wind speed, humidity, material emissivity, viewing angle, and surface reflections can all alter the imaging results [23]. In residential buildings, occupant behaviors such as window opening for ventilation, heat exhaust from air-conditioning outdoor units, shading from laundry, and temporary sunshades can also produce transient thermal anomalies [24,25]. Consequently, while a single thermographic inspection can “detect anomalies,” it may not necessarily support stable defect diagnosis.

Unmanned aerial vehicles (UAVs) equipped with IRT (UAV-IRT) have emerged as a promising technological solution for inspecting high-rise and large-scale building facades, offering enhanced accessibility and efficiency in acquiring continuous thermal imaging data from heights that are difficult for manual inspection to reach [26]. Bayomi et al. [27] applied UAV-based IRT to campus buildings in Boston and demonstrated that the method effectively identifies material aging, thermal bridges, and insulation failures with high efficiency at the building stock scale. Concurrently, advancements in 3D path planning, BIM-assisted route generation, viewpoint resampling, and multi-layer coverage path planning have improved route organization and data coverage quality for complex building surfaces [28,29,30]. For complex building facades, Huang et al. [31] proposed a BIM-supported 3D path planning method that automatically generates efficient inspection routes, significantly improving coverage completeness for geometrically intricate building surfaces. Jung et al. [32] developed a multi-layer coverage path planning algorithm that vertically segments buildings and optimizes layer-to-layer path connections, substantially reducing redundant scanning and data omissions. Nevertheless, two limitations persist in existing research. First, path planning optimization primarily targets geometric coverage, flight efficiency, and obstacle avoidance, with insufficient consideration of the coupling effects on thermal imaging quality, consistency of acquisition distance, scale comparability, and the stability of temperature-difference interpretation. Second, most thermal anomaly identification studies focus on single images or single-inspection results, lacking systematic validation of the repeatability and stability of thermal anomalies across multiple time periods and scenarios.

To address these gaps, this paper proposes an integrated diagnostic framework for facade thermal defects in old residential buildings in HSCW zones, shifting from static, single-time inspection to a multi-temporal dynamic diagnosis approach. The main contributions of this paper are as follows: (1) establishing a multi-temporal UAV-IRT data acquisition workflow for facade thermal defect diagnosis in old residential buildings, specifying key parameters including preheating time, flight distance, acquisition timing, and path organization; (2) proposing a thermal anomaly candidate region extraction method that integrates adaptive thresholding, Canny edge detection, edge traversal, and region growth, with results mapped to facade representations derived from 3D models; (3) constructing a temporal screening logic based on repeated appearance, spatial location stability, and weekday/weekend consistency to reduce temporal false positives—thermal anomalies that appear in single-moment observations but do not persist across multiple time periods and are therefore more likely to be transient environmental or behavioral responses rather than persistent construction-related thermal defects; (4) conducting case validation on three typical old residential buildings in Wuhan, developing result presentation formats oriented toward pre-retrofit diagnosis and maintenance priority assessment.

The remainder of this paper is organized as follows. Section 2 introduces the research subjects, UAV thermal imaging equipment, parameter experiments, path planning, image recognition, and temporal screening methods. Section 3 presents the equipment parameter experiment results, thermal anomaly identification, facade mapping, and case study results from typical residential communities. Section 4 discusses the improvements in diagnostic stability enabled by the multi-temporal UAV-IRT method, the engineering implications of 3D path planning, the translation of results into retrofit decisions, and issues of data reliability and privacy protection. Section 5 concludes the study and proposes directions for future research.

## 2. Materials and Methods

### 2.1. Research Subjects and Overall Framework

Located in central China, Wuhan (Hubei Province) is characterized by a typical hot-summer and cold-winter climate, with hot, humid summers and cold, damp winters. This climatic condition makes building envelopes in this region particularly susceptible to thermal defects, rendering Wuhan an ideal case study location for facade thermal defect diagnosis.

**Figure 1:**
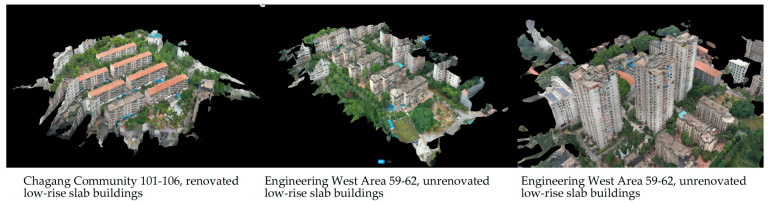
Three-dimensional models of the selected residential communities.

Three representative old residential communities on the Wuhan University campus were selected as case subjects: Chagang Community 101–106, Engineering West Area 59–62, and Engineering Building 65 (Figure 1). The field campaigns were conducted during the hottest period of summer, mainly in July 2025, and repeated measurements were arranged on both weekdays and weekends under relatively stable weather conditions. The selected buildings differ in construction age, massing, retrofit status, and facade complexity. Chagang Community has undergone partial facade renovation and is suitable for observing residual node-related anomalies and localized insulation weaknesses after renovation. Engineering West Area consists mainly of old low-rise slab residential buildings with enclosed balconies, self-built additions, moisture problems, and low-floor occlusions. Engineering Building 65 is a high-rise tower building with a relatively regular facade but with evident aging, thermal bridges, and window–wall joint anomalies. Together, the three cases cover typical engineering scenarios including low-floor occlusion, high-rise facade scanning, and comparison between renovated and unrenovated facades.

**Figure 2:**
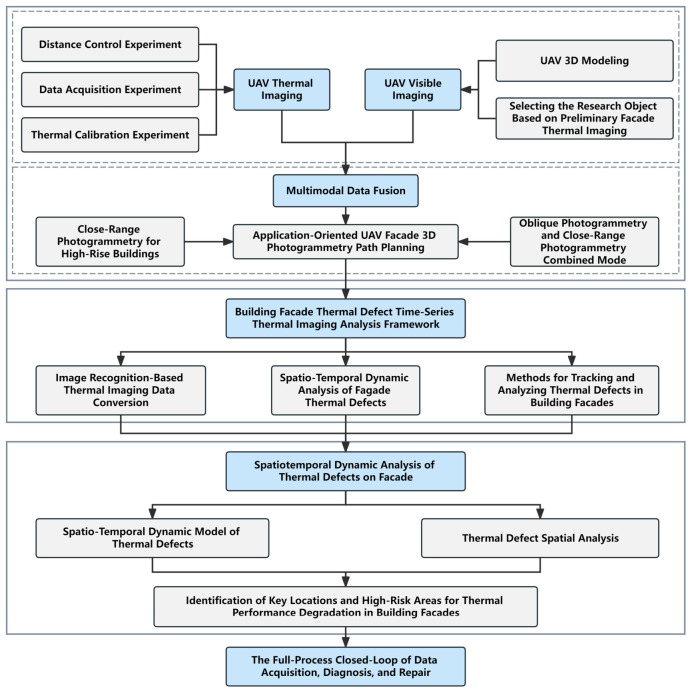
Overall workflow of the proposed multi-temporal UAV-IRT diagnostic framework.

The overall framework contains five interrelated modules, corresponding to Section 2.2, Section 2.3, Section 2.4, Section 2.5 and Section 2.6 and Figure 2. First, UAV visible-light imaging and 3D reconstruction are used to identify building height, facade orientation, occlusions, safety boundaries, and reachable viewpoints. Second, UAV thermal imaging equipment and acquisition parameters are calibrated through preheating and distance-control experiments. Third, 3D path planning is conducted for continuous facade acquisition using close-range imaging and U-shaped serpentine paths. Fourth, thermal images are preprocessed and thermal anomaly candidates are extracted through adaptive thresholding, edge detection, edge traversing, and region growth. Fifth, the extracted anomalies are mapped to facade coordinates and screened using multi-temporal observations to retain recurrent and spatially stable anomalies for field verification and retrofit-priority assessment.

### 2.2. UAV Thermal Imaging Equipment and Acquisition Parameters

This study used a DJI M30T UAV equipped with an infrared thermal camera to collect facade temperature images. The platform is suitable for inspection tasks in built-up urban environments and can maintain a relatively stable flight attitude and image quality in complex residential settings. The main parameters of the thermal camera are summarized in Table 1. Because uncooled infrared detectors exhibit thermal drift during the initial operating period, a preheating stage was set before formal acquisition, and field comparison experiments were conducted to determine the minimum stable preheating time.

In the preheating experiment, temperatures identified from thermal images were compared with thermocouple measurements following the general principle of thermographic error evaluation [33,34]. Let *T_IR,i_* denote the temperature identified from the thermal image at measurement point *i* and *T_TC,i_* denote the corresponding thermocouple temperature. The single-point measurement error is defined as:

(1) $$\Delta T_{i}=T_{IR,i}-T_{TC,i}$$

Because the experiment used four corner points for regional comparison, the regional average temperature was further calculated to reduce the influence of local fluctuation:

(2) $$T_{IR,avg}=\frac{1}{n}\sum_{i=1}^{n}T_{IR,i}$$

(3) $$T_{TC,avg}=\frac{1}{n}\sum_{i=1}^{n}T_{TC,i}$$

The regional mean measurement error is then expressed as:

(4) $$\Delta T_{avg}=T_{IR,avg}-T_{TC,avg}$$

where the overbarred terms denote the mean thermal image temperature and the mean thermocouple temperature within the selected region, respectively.

The experimental results show that equipment stability varies with weather and time of day. Under cloudy conditions at 17:00, the temperature error after 60 min of operation was approximately −2.0 °C; at 23:00, the error was approximately −3.0 °C, indicating lower stability. Under sunny conditions at 12:00 and 17:30, the error after 5–10 min of preheating could be controlled within approximately −1.0 °C. These preheating errors refer to systematic offsets in absolute temperature readings under different environmental conditions, not detector precision. These offsets affect absolute temperature values but do not compromise the detection of relative thermal contrasts between adjacent regions on the same facade, which is the basis of our anomaly segmentation. The detector’s thermal sensitivity (NETD ≤ 50 mK, Table 1) is the relevant specification for relative temperature discrimination and is substantially smaller than the absolute accuracy of ±2 °C. Considering operation efficiency, detector thermal drift, and field feasibility, this study set the preheating time before takeoff to no less than 10 min.

**Figure 3:**
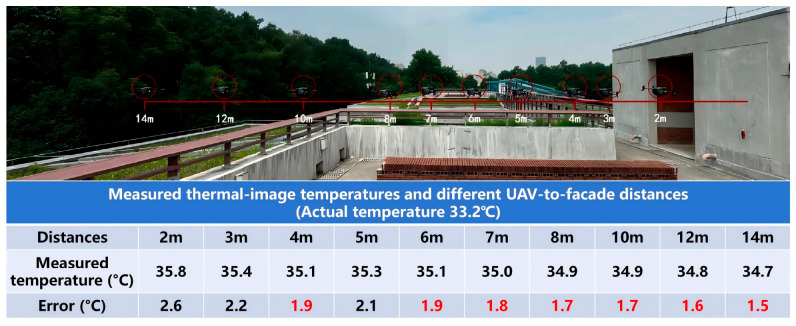
Experimental setup and results for testing UAV thermal imaging distances.

Imaging distance directly affects thermal image spatial resolution, local temperature-difference expression, and flight safety [24,29]. To analyze the influence of distance on measurement and image readability, distance-control experiments were conducted in the range of 2–14 m while keeping the camera optical axis as perpendicular to the wall as possible. Figure 3 illustrates the experimental setup and the corresponding temperature measurements. Let *d* denote the UAV-to-facade distance; the average measurement error under distance *d* can be written as:

(5) $$\Delta T(d)=T_{IR}(d)-T_{TC}$$

The observed decrease in measurement error with increasing distance can be attributed to the spatial integration effect of the infrared detector. At close range, the IFOV resolves a very small surface area, making the measurement sensitive to local material heterogeneities. As distance increases, the measurement integrates a wider area and averages these microscale thermal fluctuations.

The results show that when the distance is too short, the single image covers only a limited facade area and the component scale is difficult to unify; when the distance is too long, spatial resolution decreases and the boundaries of local thermal anomalies become weakened. The range of 8–10 m provides a relatively balanced condition among measurement stability, image readability, and flight safety, with a temperature-difference fluctuation of approximately 1.7 °C. The 1.7 °C fluctuation refers to the range of measurement errors observed across multiple repeated acquisitions at distances within the 8–10 m interval. Readings at 8 m, 9 m, and 10 m all fell within approximately 1.7 °C (varying from roughly 1.5 °C to 1.9 °C), indicating stable and repeatable measurements in this range. Therefore, the subsequent case acquisition controlled the UAV-to-facade distance within 8–10 m and maintained a near-orthogonal viewing direction wherever possible. The final control settings for UAV thermal imaging are summarized in Table 2.

For all facade acquisitions, the emissivity was set to 0.95, which is representative of common building materials (brick, concrete, and painted surfaces) in the long-wave infrared range (8–14 μm). The reflected apparent temperature was set to 20 °C. Both parameters were kept strictly uniform across all acquisition batches and buildings to ensure data comparability.

The M30T is powered by two TB30 intelligent flight batteries (5880 mAh, 26.1 V, and 131.6 Wh each), providing a maximum flight time of approximately 45 min per battery set under standard operating conditions. In practice, for the multi-temporal acquisition campaigns conducted in this study, each battery set was used for approximately 30 min of effective flight time per building scanning session (from 100% to approximately 20% remaining capacity), with battery replacement scheduled between sessions.

### 2.3. 3D Path Planning and Continuous Facade Acquisition

Traditional two-dimensional oblique routes tend to generate local occlusion, scale inconsistency, and coverage gaps in facade thermal imaging, especially in old residential communities where trees, overhead pipelines, parking sheds, protruding balconies, and adjacent buildings significantly affect line-of-sight accessibility. Based on visible-light 3D reconstruction, this study divided each target facade into continuously scannable facade units and generated candidate viewpoints around each unit. The objective of path planning was not to minimize flight distance, but to maintain a relatively consistent imaging distance and viewing angle within safety boundaries, thereby reducing temperature-interpretation bias caused by scale differences [28,29,30,31,32,35,36]. Table 3 summarizes the 3D-model-assisted path planning workflow.

The flight paths were planned and executed using a combination of software tools. First, DJI Pilot 2 V14was used for visible-light oblique photogrammetry to generate the 3D reference model. Then, DPGO (DuoPuZhiHang) V2.3.6 was used for close-range path planning, which generates facade-adaptive waypoint missions based on the 3D model geometry. Finally, the planned missions were executed via DJI Terra, which also handled the visible-light model reconstruction and thermal image registration. In terms of acquisition time, each complete facade scanning session for one residential community (covering the target facades within a single time slot) required approximately 30 min of effective flight time, corresponding to one battery set consumption from 100% to 20% remaining capacity. For a complete multi-temporal campaign covering three time slots (9:00, 14:00, and 17:00) on both a weekday and a weekend, approximately six flight sessions were required per building.

**Figure 4:**
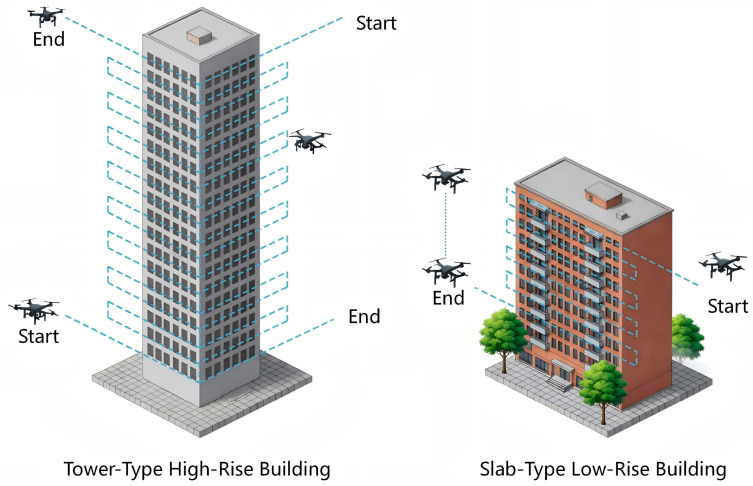
U-shaped serpentine close-range photogrammetry path for facade thermal imaging.

**Figure 5:**
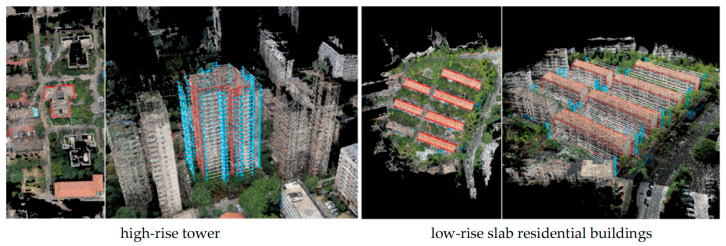
3D path planning examples.

In route organization, a U-shaped serpentine path was adopted according to a “layered scanning–horizontal advancement–vertical transition” logic. The UAV moved back and forth along the horizontal direction of the facade and then shifted vertically after completing one floor or one height band, forming a continuous scanning sequence for the entire facade (Figure 4). For high-rise residential buildings, close-range vertical layered scanning was mainly used; for low-rise or heavily occluded buildings, oblique supplementary imaging and local viewpoint adjustment were combined to improve the completeness of low-floor, corner, and occluded areas (Figure 5).

### 2.4. Thermal Image Preprocessing and Anomaly Recognition

**Figure 6:**
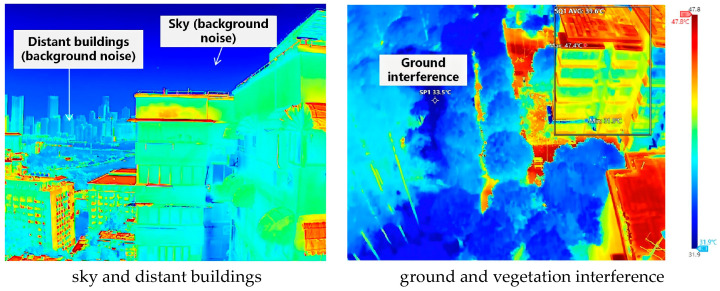
Removal of non-target regions in thermal image preprocessing.

Raw UAV thermal images usually contain non-target regions such as sky, ground, trees, and adjacent structures. These regions do not belong to the target facade and often exhibit large temperature differences, which can interfere with threshold segmentation and anomaly identification [4,5,13,23]. Therefore, this study first cropped the target facade and removed background interference (Figure 6), retaining only building surface regions relevant to the inspection task. The thermal palette and temperature display range were then unified to make images from the same and different acquisition batches comparable. Local contrast was further enhanced to improve the readability of walls, window frames, balconies, floor-slab lines, and potential thermal anomalies.

After preprocessing, the next task was to automatically identify and spatially localize thermal anomaly regions. It should be noted that this study does not employ deep learning-based object detection models. Instead, we adopt a multi-stage traditional image processing pipeline consisting of global adaptive thresholding, Canny edge detection, edge traversal, and seeded region growth. This pipeline is fully deterministic and does not require training data. Based on conventional image-processing methods, this study developed a multi-stage recognition workflow consisting of thermal anomaly pre-screening, boundary extraction, spatial refinement, and region segmentation. The workflow combines threshold filtering with spatial constraints to identify anomalies from coarse to fine. Because thermal environments vary significantly among facades, fixed temperature-difference thresholds may lead to poor generalization. Therefore, this study adopted a multi-stage mechanism consisting of adaptive thresholding, Canny boundary extraction, edge-traversing refinement, and region-growing segmentation. The output of this section is a thermal anomaly candidate mask for subsequent annotation and classification; it is not directly equivalent to a final defect-type diagnosis.

#### 2.4.1. Global Adaptive Threshold Calculation

The key task in facade thermal defect detection is to capture abrupt and abnormal temperature variations in thermal images. Since fixed-threshold methods cannot be generalized to defects of different severities and to changing facade environments, this study adopted an adaptive thresholding method based on the temperature histogram [37]. Let the acquired UAV thermal image be a two-dimensional matrix *I*(x,y), in which each pixel records an absolute temperature value. The algorithm first constructs a temperature histogram with M bins. The bin containing extreme temperature values is defined as the abnormal temperature bin *B_leak_*, and the farthest relative bin in the histogram is defined as the reference bin *B_ref_*.

To ensure the statistical reliability of threshold selection, the model dynamically evaluates the distribution of pixels in each histogram bin. The pixel proportion of the reference bin is required to exceed 10% of all pixels, while the abnormal temperature bin, which corresponds to local defect regions, must account for less than 15%. These empirical proportions were adjusted according to multiple facade thermal image samples to prevent the threshold from being dominated by large background temperature zones. When the statistical distribution does not meet these constraints, the number of bins is increased iteratively. Under the final converged histogram, the difference threshold *T_diff_* and defect threshold *T_defect_* are extracted as follows:

(6) $$T_{diff}=\max(B_{M-1})-\min(B_{M-1})$$

(7) $$T_{defect}=Boundary(B_{defect},B_{adjacent})$$

where $B_{M-1}$ denotes the second-to-last histogram bin, and width ($B_{M-1}$) represents the temperature span of that bin. The function boundary (·) extracts the scalar boundary value between two adjacent bins, and $B_{adjacent}$ denotes the histogram bin adjacent to the defect bin.

#### 2.4.2. Initial Physical Boundary Extraction Using the Canny Algorithm

After global threshold calculation, the algorithm enters the spatial feature extraction stage for thermal anomaly candidates. Two key assumptions underlie this approach. First, thermal anomalies are assumed to manifest as local temperature deviations that are statistically distinguishable from the background facade temperature distribution. Second, true thermal defects are assumed to exhibit spatial consistency across multiple observations, which is why temporal screening is introduced as a post-processing verification step rather than relying on single-image classification. Candidate regions in thermal images usually appear as patches with temperatures significantly different from their surroundings. Because a thermal image is essentially a two-dimensional scalar temperature matrix, the abrupt temperature change at the boundary between a defect region and a normal region can be treated mathematically as an image edge. Therefore, edge detection can be used to identify sharp intensity transitions in the temperature matrix. Based on this principle, the Canny edge detector was introduced to extract initial thermodynamic boundaries [38].

The Canny operator is an optimal edge detector with a multi-step processing mechanism. Its objective is to localize true image edges with a low error rate [38]. The procedure includes Gaussian smoothing, gradient calculation, non-maximum suppression, and double-threshold hysteresis. First, a two-dimensional Gaussian smoothing filter is convolved with the input thermal image to suppress high-frequency sensor noise. The Gaussian kernel is expressed as:

(8) $$G(x,y)=\frac{1}{2\pi \sigma^{2}}e^{-\frac{x^{2}+y^{2}}{2\sigma^{2}}}$$

Then, finite-difference operators are used to calculate gradients in the horizontal (x) and vertical (y) directions of the smoothed image, and the gradient magnitude and direction of each pixel are obtained as:

(9) $$M(x,y)=\sqrt{G_{x}^{2}+G_{y}^{2}}$$

(10) $$\theta (x,y)=\arctan\left(\frac{G_{y}}{G_{x}}\right)$$

After the gradient field is obtained, non-maximum suppression is applied along the gradient direction to refine edge lines. A double-threshold hysteresis process then connects strong edges and removes weak noisy edges. For facade thermal anomaly detection, a relatively aggressive low-threshold strategy was used to retain as many potential anomaly boundaries as possible. The standard deviation of the Gaussian smoothing filter was set to sigma = 2, and the normalized low and high hysteresis thresholds were set to 0.04 and 0.20, respectively. These parameters were determined through repeated testing on facade thermal image samples. Although such a low-threshold strategy may introduce structural textures such as normal window frames, these false edge candidates are subsequently removed through the context-aware edge-traversing mechanism.

#### 2.4.3. Spatial Feature Refinement and Region Growth Based on Edge Traversing

Although Canny edge detection can capture local physical boundaries, the initial edge set often includes many non-thermal structural textures. To remove this spatial noise, a context-aware edge-traversing mechanism was introduced. For each visited edge pixel, a one-dimensional neighborhood vector is constructed perpendicular to the local edge orientation. The model performs nonlinear aggregation and extreme-value retrieval within the neighborhood. The key decision rule is expressed as [39]:

(11) $$\Delta T=\max(V_{p})-\min(V_{p})>T_{diff}$$

(12) $$\min(V_{p})<T_{leak}$$

where $T_{max}$ and $T_{min}$ denote the maximum and minimum temperatures within the orthogonal neighborhood vector, respectively. Only when the local temperature contrast exceeds the adaptive difference threshold and the neighborhood extreme value exceeds the defect threshold is the edge pixel and its corresponding extreme-temperature point retained as a true defect-boundary feature point.

After these two constraints are satisfied, the model records the temperature extreme point with the largest deviation and uses the retained high-confidence feature points as seeds for region growth [39]. During region growth, the neighborhood-merging temperature-difference threshold was set to 0.1 °C to suppress boundary spillover. This threshold is based on the relative temperature contrast between adjacent pixels, not absolute temperature accuracy. The thermal camera used in this study has an NETD of ≤50 mK (Table 1), which provides sufficient thermal sensitivity to resolve relative temperature differences at the 0.1 °C scale. While the absolute temperature accuracy is ±2 °C, this systematic bias affects global temperature readings but does not compromise local contrast-based segmentation decisions. A disk-shaped structural element with a radius of 3 pixels was then used for morphological closing to fill small holes inside the detected regions, resulting in more complete local anomaly segmentation. For batch processing of multi-temporal acquisitions, the processing time per image was acceptable within our workflow. However, a systematic benchmark was not conducted, as the proposed workflow operates offline (post-acquisition analysis) rather than in real time. Future work will include a quantitative performance evaluation when the pipeline is optimized for larger-scale deployment.

Thermal anomalies in building facades exhibit diverse temperature distributions, shapes, and heat-transfer characteristics. To facilitate interpretation of the thermal anomalies identified in this study, we define four representative categories according to typical thermal mechanisms and engineering interpretability (Table 4). This classification framework is qualitative and descriptive, based on the established literature and thermal physics principles. It is used as a reference for manual interpretation of thermal patterns in the Section 4, rather than as an automated classification output. Borderline samples were allowed to be marked as uncertain during review to avoid forced categorization.

During annotation, the classification of each anomaly was determined by jointly considering temperature distribution, boundary morphology, local thermal contrast, and its relationship with facade components. Regions with stable and relatively uniform temperature deviation were usually regarded as thermal defect candidates, localized temperature spikes around joints were interpreted as possible thermal bridges, sharp temperature variations around linear features were associated with cracks or joints, and irregular thermal regions were treated as uneven thermal distributions that may be related to material aging, moisture, or construction quality. This procedure was based on the criteria in Table 4 and subsequent field interpretation.

### 2.5. 3D Facade Mapping and Temporal Stability Screening

**Figure 7:**
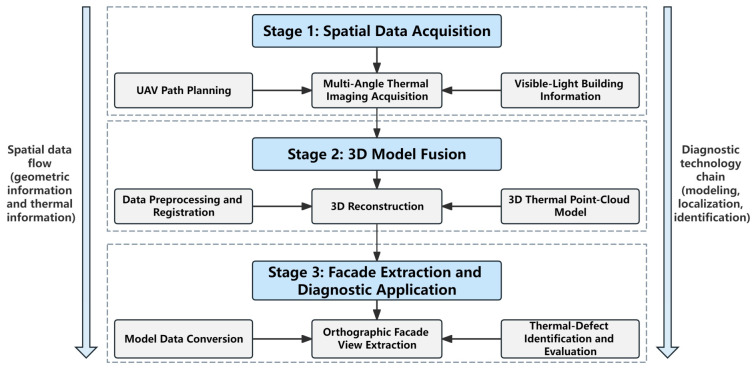
Three-stage facade extraction and spatial mapping workflow based on UAV dynamic thermal imaging.

If thermal anomalies remain only in individual two-dimensional thermal images, their spatial attribution is often unclear; it is difficult to determine the corresponding building, floor, bay, or construction joint. To solve this problem, thermal imaging results were combined with 3D reconstruction data. DJI Terra and related tools were used to generate 3D building models, and thermal textures or registered thermal image results were projected to facade-oriented views, producing thermal facade representations for spatial interpretation (Figure 7). The anomaly regions identified in two-dimensional images were registered and superimposed onto complete facade views so that they could be interpreted consistently at the level of floors, bays, window–wall joints, and wall components [31,35,36].

To reduce the influence of environmental disturbance and occupant behavior, temporal stability screening was further applied. Multi-temporal acquisition included three time points on the same day—09:00, 14:00, and 17:00—and one weekday and one weekend were selected within the same week under similar weather conditions. The morning period was used to observe the initial temperature distribution under lower solar radiation; the noon period was used to observe explicit anomalies enhanced by solar radiation and outdoor heat; and the afternoon period was used to observe the more stable response generated by structural heat storage and thermal inertia after solar radiation weakened.

**Figure 8:**
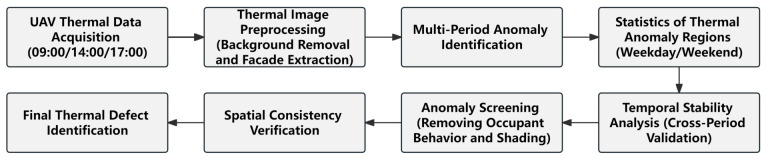
Temporal thermal defect screening workflow based on repeated UAV-IRT observations.

Based on the facade mapping results, a temporal analysis workflow was constructed to improve the stability and interpretability of anomaly identification. Instead of interpreting each thermal image independently, the workflow places anomaly results from different time points and date types into a common analytical framework and screens them according to anomaly persistence, spatial-position stability, and temperature-response consistency. Its purpose is to retain anomalies that recur across multiple periods and contexts while removing transient anomalies that appear only at a specific time or under a specific occupant or environmental condition. The overall procedure is shown in Figure 8.

Anomaly persistence was used to determine whether the same facade location repeatedly appeared as a thermal anomaly in multiple observations. For the i-th anomaly region, its occurrence frequency $P_{i}$ is calculated as follows:

(13) $$P_{i}=\frac{n_{i}}{N}$$

where $P_{i}$ is the persistence ratio of the *i*-th anomaly, $n_{i}$ is the number of observations in which the region is identified as anomalous, and N = 6 in this study (3 time slots × 2 date types: weekday and weekend). A persistence threshold of *P_i_* ≥ 2/3 (i.e., appearing in at least 4 out of 6 observations) is applied to retain an anomaly as temporally stable. This threshold was selected to require the anomaly to appear across both weekday and weekend conditions and in at least two different diurnal time slots, ensuring robustness against single-day or single-moment disturbances. In interpretation, a region that recurs across multiple observations, especially under both weekday and weekend conditions, is regarded as having stronger persistence. To avoid misclassification caused by a single threshold, persistence was used together with spatial stability, temperature-response consistency, and construction-related evidence.

Different facade materials (glass, metal AC units, painted surfaces, and brick) have distinct emissivities and reflectivities. However, our analytical focus is specifically on thermal anomalies on opaque wall surfaces, not on evaluating window glass itself. Thermal anomalies “around windows” in our analysis refer to the wall–window interface and surrounding wall areas, not the glass surface. To reduce specular reflections from glass, we adopted two strategies: (a) maintaining the camera optical axis as perpendicular to the facade as possible during acquisition; (b) filtering out anomalies that appeared only on glass surfaces or exhibited strong specular reflection patterns during the temporal screening process, as such patterns typically lack spatial consistency across multiple time periods.

### 2.6. Data Organization and Evaluation Metrics

To ensure comparability among multi-temporal data, all collected images were organized according to the hierarchy of building–facade–date–time period–image number. Each thermal image recorded the acquisition time, building, facade orientation, flight distance, camera angle, and weather condition. After preprocessing, a unified image file, anomaly-mask file, and façade mapping result were generated. For the same building facade, the morning, noon, and afternoon acquisitions, as well as weekday/weekend contexts, were included in the same comparison group. This data organization avoided fragmentation caused by isolated image interpretation and facilitated subsequent batch statistics and model training.

The evaluation included three levels. The first was acquisition quality, including whether the image fully covered the target facade, whether severe occlusion or reflection existed, whether the imaging distance remained within the set range, and whether the image was blurred or overexposed. The second was recognition output, including the number, area, shape, boundary clarity, and constructional correspondence of thermal anomaly regions. The third was temporal stability, including recurrence count, cross-date consistency, weekday/weekend consistency, and spatial deviation on the facade. Because the objective of this study is pre-retrofit diagnosis rather than pixel-level segmentation accuracy, the emphasis was not a single IoU value but whether the results could be stably localized at the facade scale and could provide useful evidence for field verification.

For spatial consistency, facade coordinates and building construction units were used as the primary basis rather than pixel displacement alone. Slight changes in UAV viewpoint and imaging scale are unavoidable in repeated acquisition; if pixel coordinates alone are used, a stable anomaly around the same construction joint may be misinterpreted as a positional change. Mapping anomaly regions to facade views allows interpretation according to floors, bays, window openings, floor-slab lines, and wall joints, which is more consistent with building-diagnosis practice.

Thermal anomaly interpretation followed a combined logic of image features, spatial location, temporal behavior, and field construction evidence. Image features provide clues regarding temperature contrast and shape, spatial location provides constructional attribution, temporal behavior provides evidence of stability, and field construction evidence supports causal interpretation. Only when these four types of evidence support each other is an anomaly considered a high-confidence thermal defect candidate; if an anomaly is obvious in a single image but temporally unstable or inconsistent with construction logic, it requires remeasurement or should be treated as interference.

## 3. Results

### 3.1. Parameter Experiment Results and Acquisition Strategy

The preheating experiment indicates that uncooled infrared thermal imagers exhibit obvious thermal drift during the initial operation period, and direct acquisition without preheating can affect the consistency of temperature-field interpretation. As the operating time increased, the measurement results gradually stabilized. Combining cloudy, night-time, and sunny experiments, the error under sunny conditions at 12:00 and 17:30 could be controlled within approximately −1.0 °C after 5–10 min of preheating. Therefore, no less than 10 min of preheating before formal operation was adopted as a basic requirement. This parameter is intended not to pursue absolute temperature accuracy, but to maintain a relatively stable thermal response across multiple data batches.

The distance experiment further shows that imaging distance is a key factor affecting the usability of UAV-IRT data. At a short distance of 2–3 m, local details were abundant but facade coverage was incomplete; therefore, it was difficult to obtain continuous representation at the floor and bay scales. At 4–7 m, image readability improved, but scale fluctuation remained. At 8–10 m, anomaly boundaries, facade coverage, and measurement stability were relatively balanced. When the distance further increased, local anomaly boundaries became blurred. Thus, the optimal distance for facade thermal defect diagnosis is jointly constrained by temperature stability, spatial resolution, component recognition, and flight safety.

Based on these results, the case acquisition adopted the following unified settings: preheating for no less than 10 min before takeoff, a UAV-to-facade distance of 8–10 m, a near-orthogonal camera angle to the facade, field acquisition during relatively stable weather in the summer days of July and August, and repeated observations at 09:00, 14:00, and 17:00 on both a weekday and a weekend. These settings provided a consistent data boundary for subsequent multi-temporal comparison.

### 3.2. Thermal Anomaly Identification and Facade Spatial Representation

After preprocessing, threshold-based pre-screening, edge extraction, and region growth, thermal anomaly candidate regions were segmented from the original thermal images. Compared with direct manual interpretation, automated candidate extraction reduces dependence on single-image visual color differences and enables subsequent analysis based on anomaly number, area, location, and shape. Four main spatial patterns were observed: discrete point or node-like anomalies around window frames, door frames, and air-conditioner openings; linear or band-like anomalies along floor-slab lines, balcony edges, window–wall joints, and wall seams; patch-like or clustered anomalies associated with insulation detachment, aging, or wall moisture; and composite anomalies where point, linear, and patch patterns overlap within the same facade area.

**Figure 9:**
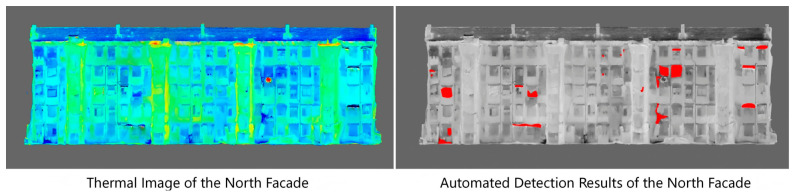
Comparison of facade thermal imaging and thermal anomaly identification results.

3D facade mapping improves the spatial interpretability of recognition results. After facade registration, anomaly regions in individual thermal images can be located on specific floors, bays, and construction joints. As shown in Figure 9, the left thermal facade representation provides a continuous temperature background, while the right image with red overlays presents the distribution of thermal anomalies at the facade scale. For energy-efficiency retrofitting, this expression is more useful than isolated thermal screenshots because retrofit decisions are usually organized by building, facade, floor, and construction detail.

### 3.3. Multi-Temporal Thermal Anomaly Screening Results

Multi-temporal comparison shows that the number and spatial distribution of thermal anomalies on the same facade varied significantly among different time points, as summarized in Table 5. For a typical facade, 9 anomalies were detected at 09:00 on a weekday, 32 at 14:00, and 21 at 17:00; on the weekend, the counts were 13 at 09:00, 34 at 14:00, and 40 at 17:00. Noon produced more anomalies due to enhanced solar radiation, window opening, air-conditioner operation, and rapid local surface-temperature changes. In the afternoon, some transient anomalies disappeared, whereas anomalies associated with wall heat storage, insulation weakness, and construction joints were more likely to persist.

**Figure 10:**
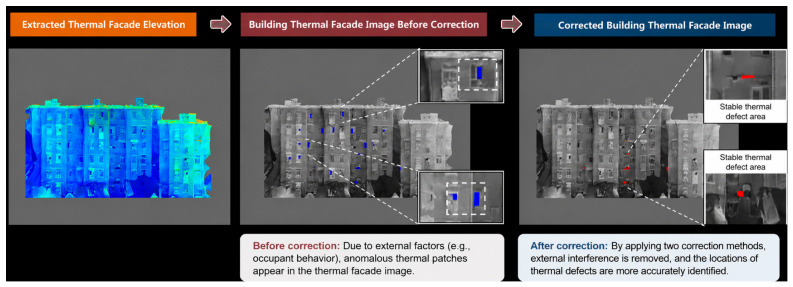
Comparison of facade thermal imaging results before and after temporal screening.

These results indicate that the number of anomalies obtained from a single acquisition should not be directly equated with the number of structural thermal defects. Some anomalies appear only at noon or under a single context and have strong randomness, while others repeatedly appear across multiple periods and are spatially consistent with window–wall joints, floor-slab lines, or moisture-affected wall areas. After temporal screening, transient anomalies are removed or downgraded, and the retained regions are more suitable for field verification and retrofit-priority assessment. Figure 10 compares the facade thermal imaging results before and after temporal screening.

From the perspective of building type, low-rise slab buildings and high-rise tower buildings impose different requirements on UAV-IRT. Low-rise slab buildings are more affected by surrounding trees, vehicles, temporary sheds, and auxiliary facilities, so supplementary imaging and oblique-view correction are often necessary for low-floor areas. High-rise tower buildings have more continuous facades and are suitable for regular layered scanning, but they require stricter control of flight stability, safety boundaries, and imaging distance. The interpretation of renovated and unrenovated buildings also differs: renovated facades require more attention to repaired areas, node closures, and localized aging, whereas unrenovated facades may reflect overall envelope thermal performance deficiencies.

The morning, noon, and afternoon periods are not simple repeated samples; rather, they correspond to different thermal boundary conditions. The morning period, with weaker solar radiation, is suitable for observing baseline temperature differences; the noon period, with stronger thermal excitation, can amplify some surface anomalies but also introduces stronger radiation and occupant-behavior interference; and the afternoon period, when radiation weakens, better reveals thermal inertia and structural heat storage. These time slices together capture the appearance, enhancement, and decay of anomalies, which is useful for judging persistence.

### 3.4. Diagnostic Results of Typical Residential Communities

Across the three residential communities, the number of anomalies retained after temporal screening differed according to building type and retrofit status (Table 6). For Chagang Community 101–106, the retained anomaly counts were 26, 15, 14, 38, 29, and 24, respectively, indicating that local node-related and wall-surface thermal weaknesses remained after previous facade renovation. For Engineering West Area 59–62, the counts were 19, 16, 26, and 40, respectively, and the anomalies in unrenovated buildings were more prominent around wall patches, enclosed balconies, leakage cracks, and moisture-affected regions. For Engineering Building 65, the north/south facades contained 69 anomalies, while the east/west facades contained 43, suggesting concentrated recurrent anomalies around window–wall joints, floor-slab lines, and aged exterior wall areas of the high-rise tower.

To illustrate the screening process, we provide a worked example using Engineering West Area 61′s North Facade. Across the six observation sessions, thermal anomaly counts per session ranged from 13 to 40 (Table 5). Applying the temporal persistence criterion (*P_i_* ≥ 2/3) retained regions that appeared consistently across both weekday and weekend conditions. Spatial stability screening (±1 m vertical tolerance across repeated observations) further removed regions whose positions shifted noticeably between sessions. Finally, the construction-relevance filter excluded anomalies that lacked spatial correspondence to identifiable construction elements such as window frames, slab lines, or wall joints. After this three-stage screening process, 26 regions were retained as persistent thermal anomalies for this facade (Table 6). The retained regions were then mapped to the facade representation.

**Figure 11:**
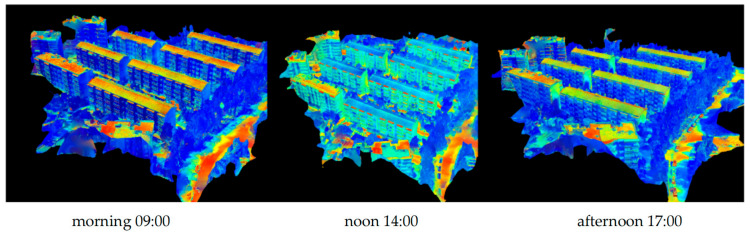
Three-period 3D infrared thermal imaging models of Chagang Community.

Figure 11 further illustrates the temporal characteristics of the thermal field in Chagang Community. Spatially, renovated facades were not free from thermal anomalies; their remaining problems mainly appeared around window frames, local repaired wall areas, and joints. Unrenovated facades more often exhibited patch-like or band-like distributions, reflecting insufficient envelope thermal performance, missing insulation continuity, or material aging. In high-rise buildings, regular facades facilitate continuous scanning and mapping, but the large number of window–wall joints, continuous slab lines, and long-term exposure to sun and rain can produce recurrent node-like and linear anomalies.

## 4. Discussion

### 4.1. Stability and Reliability of the Multi-Temporal UAV-IRT Method

It should be emphasized that summer daytime thermal anomalies are not direct measurements of winter heat loss. Rather, they represent surface temperature responses that, when observed persistently across multiple time periods, may indicate localized variations in thermal inertia, construction continuity, or material condition. Under summer daytime conditions with strong solar radiation, facade surface temperatures are primarily driven by absorbed solar gain, material thermal inertia, and local convective cooling, rather than by indoor-to-outdoor heat conduction. However, thermal anomalies observed persistently under these conditions can still provide diagnostically useful information through two mechanisms: (a) thermal inertia contrast—defective or degraded insulation reduces thermal mass effectiveness, causing faster surface temperature rise under solar loading and slower cooling after solar peak; (b) construction continuity—thermal bridges, cracks, and material interfaces create localized thermal gradients that manifest as persistent spatial patterns regardless of the dominant heat-flow direction. These mechanisms are complementary to, rather than substitutes for, winter heat-loss measurement.

The results demonstrate the necessity of multi-temporal observation for diagnosing facade thermal defects in old residential buildings. Conventional single-time IRT commonly relies on a temperature-difference image acquired at one moment; when a high- or low-temperature region appears, it can easily be interpreted directly as a thermal defect. In real residential environments, however, thermal anomalies are controlled not only by construction defects but also by short-term occupant behavior and external environmental conditions. Window opening can produce local temperature changes near openings, air-conditioner exhaust can generate high-temperature patches, and clothes drying or tree shading can produce low-temperature or irregular boundary anomalies. These anomalies are often short-lived, spatially unstable, and weakly related to construction details.

The value of multi-temporal screening lies in shifting interpretation from instantaneous anomaly discovery to stable anomaly confirmation. When an anomaly recurs across multiple time periods and usage contexts and is spatially associated with a construction joint, it is more likely to have structural or material causes. Conversely, if an anomaly appears only at noon or under a single context and is related to window opening, shading, or air-conditioner exhaust, it should be treated cautiously before being included in a retrofit list. The method cannot replace field-opening inspection or material testing, but it can improve the credibility of thermal imaging results before they enter engineering decisions.

Because facade thermal imaging of old residential buildings involves real living environments, data reliability and privacy protection are both important components of the method. Four reliability-control measures were adopted: sensor preheating and distance experiments to reduce thermal drift and scale-related bias; unified acquisition time, palette range, and imaging distance to improve comparability among data batches; 3D path planning to reduce occlusion, oblique-viewing errors, and discontinuous coverage; and temporal screening to reduce random anomalies caused by environmental fluctuations and occupant behavior. These measures support the transformation of thermal images from visual evidence into comparable data.

From the perspective of privacy protection, UAV inspection of residential facades may capture windows, balconies, clothes drying, or traces of occupant activity. In data processing and manuscript presentation, images containing identifiable personal or household information were avoided or weakened. The study focuses on facade thermal conditions and construction elements and does not conduct individualized analysis of occupant behavior. The displayed figures emphasize whole facades, local construction features, and anomaly masks, avoiding clear faces, indoor spaces, or identifiable personal items. If the method is applied to urban renewal or property inspection, further procedures concerning flight approval, public notification, data anonymization, access control, and result-use boundaries should be established.

### 4.2. 3D Path Planning, Facade Mapping, and Diagnostic Translation

The advantage of UAV platforms lies not only in reaching high positions, but also in producing repeatable, continuous, and relatively scale-consistent acquisition through path control. In building thermography, imaging distance, viewing angle, and coverage continuity directly affect anomaly boundaries and temperature-difference comparability. If images are acquired from very different distances and angles, even the same recognition algorithm may generate biased outputs due to scale change. In this study, 3D-model-assisted path design integrates viewpoint generation, occlusion checking, and U-shaped serpentine route organization, helping to improve data consistency among facade zones.

Facade mapping further solves the problem that anomalies are located in image space but not yet attributed to building construction. Thermal results can support maintenance planning and energy-efficiency retrofitting only after they are translated into facades, floors, bays, and construction nodes. For property managers and design teams, the required output is the repair priority of a specific building, facade, floor, and construction detail, rather than isolated thermal image screenshots. Therefore, 3D path planning and facade mapping jointly form the bridge from UAV-IRT data acquisition to engineering diagnosis.

Translating thermal imaging results into retrofit actions is a key objective of this study. In images, anomalies are expressed as temperature differences; in engineering application, they must be interpreted as treatable construction objects, maintenance objects, and management objects. Based on the case results, the engineering translation of anomalies can be divided into three levels. The first is node-level repair, corresponding mainly to stable anomalies around window frames, door/window openings, air-conditioner holes, and window–wall joints. Typical measures include adding weather-resistant sealant, replacing aged sealing strips, repairing insulation returns around frames, and improving local air-tightness. The second is local construction repair, corresponding mainly to linear anomalies around slab lines, balcony slabs, wall joints, rainwater pipes, and cracks, with emphasis on restoring insulation continuity, waterproofing continuity, and node integrity. The third is systematic retrofit, corresponding mainly to large stable patch-like anomalies on unrenovated facades, which usually indicate insufficient overall envelope thermal performance and require zonal or whole-facade insulation upgrading rather than isolated repair.

In the case buildings, Chagang Community had undergone facade renovation but stable anomalies still appeared around window–wall joints, local repaired areas, and wall seams. This indicates that previous renovation does not necessarily eliminate all thermal weak points, and subsequent maintenance should focus on node details and localized deterioration. The unrenovated buildings in Engineering West Area exhibited broader anomaly distributions, including window openings, wall patches, and moisture-crack zones, suggesting that their retrofit strategy should not be limited to scattered repair but should be integrated with overall envelope-performance improvement. Engineering Building 65, although geometrically regular and suitable for continuous UAV scanning, showed repeated anomalies along window–wall joints and slab lines due to long-term solar radiation, weathering, and material aging.

To improve usability, anomaly regions should be expressed according to stability, constructional relevance, and retrofit urgency. Stability indicates whether an anomaly recurs across time periods; constructional relevance indicates whether it corresponds to window–wall joints, slab lines, balcony slabs, cracks, or moisture traces; and retrofit urgency considers anomaly area, recurrence frequency, resident feedback, and visible field damage. Highly stable anomalies with clear constructional relevance should be prioritized for field verification. Large but unstable anomalies should be remeasured under controlled weather and usage conditions. Anomalies clearly caused by air-conditioner exhaust, window opening, clothes drying, or temporary shading should be excluded from the structural-defect list to avoid unnecessary construction investment.

### 4.3. Comparison with Existing Studies and Method Boundaries

Compared with existing IRT studies, the contribution of this work is not a single new sensor or an entirely new recognition algorithm, but the integration of sensing, spatial path planning, image processing, and temporal verification into a complete diagnostic chain. Previous studies have demonstrated the effectiveness of IRT in identifying thermal bridges, moisture, cladding detachment, insulation defects, and window-related air leakage, and UAV-IRT has also been applied to building envelope assessment, calibration, and large-scale inspection. However, for old residential facades with complex occupant behavior, heterogeneous construction conditions, and diverse occlusions, imaging efficiency alone is insufficient to ensure diagnostic reliability. This study combines distance and angle control in path planning, candidate extraction in image recognition, facade attribution in 3D models, and stability verification in temporal data, thereby forming an engineering-oriented workflow.

In addition, this study emphasizes the constraining role of parameter experiments in field acquisition. Sensor preheating, imaging distance, and acquisition timing are often treated as operational experience, but they directly influence data stability and comparability. By translating these factors into explicit control parameters, UAV-IRT results can be more readily compared across buildings, facades, and acquisition batches. This is meaningful for future thermal defect databases and batch inspection of old residential buildings.

This study also has methodological boundaries. First, thermal images mainly reflect surface temperature responses and cannot directly reveal internal wall construction; final defect confirmation still requires field verification. Second, low-floor areas are often occluded by trees, vehicles, and auxiliary structures, so purely aerial acquisition may leave blind spots; future studies can combine UAV imaging with ground-based handheld thermography, visible-light images, and laser point clouds for air-ground collaborative inspection. Third, this study focuses on summer high-temperature conditions; winter heating conditions, night-time stable conditions, and other climate zones require further verification. Finally, the stability scoring framework remains rule-based, and future work should calibrate weights using larger samples and field-opening results while introducing learning-based models for automatic recognition and classification.

The retrofit suggestions proposed in this study do not replace construction drawings or material testing; rather, they provide technical clues for pre-retrofit screening and field verification. Thermal imaging results should be combined with visible-light crack photographs, moisture traces, indoor resident feedback, property-maintenance records, and necessary local opening inspections to form final repair plans. For insulation delamination, wall moisture, and crack leakage, surface temperature fields alone are insufficient to fully determine internal construction conditions; UAV-IRT should therefore be integrated with tapping inspection, moisture content testing, close-range visible-image recognition, and BIM component information.

### 4.4. Limitations and Future Work

Although this study establishes a relatively complete workflow that combines UAV dynamic thermal imaging, multi-temporal observation, 3D path planning, and facade anomaly recognition, several limitations remain and should be addressed in future work.

At the data-acquisition level, low-floor facade areas in old residential buildings are easily occluded by trees, vehicles, pipelines, and auxiliary components. Although 3D path planning and supplementary imaging improved data coverage, acquisition completeness in complex street-block environments still needs improvement. Future studies can combine UAV-based thermal imaging with ground-based thermal imaging, handheld devices, or fixed monitoring points to form an air–ground collaborative acquisition mode. In addition, this study mainly focuses on summer high-temperature conditions, and its applicability under winter heating conditions, night-time stable conditions, and other climate zones requires further validation.

At the data-fusion and automation levels, this study uses thermal infrared data as the core and combines visible-light images and 3D models for auxiliary analysis, but BIM, LiDAR, energy monitoring, and material-aging models have not yet been fully integrated. Future work can proceed in three directions: first, improving automated and intelligent analysis by using deep learning to integrate data acquisition, anomaly recognition, and diagnostic recommendations; second, integrating thermal images with visible-light images, LiDAR point clouds, and BIM models to improve the recognition of complex construction details and hidden defects; and third, constructing engineering case libraries for different building types and climate regions to support more targeted retrofit strategies.

At the privacy-protection and standardization levels, UAV inspection of residential facades may collect sensitive information. This study avoided or weakened images containing identifiable personal or household information. Future applications should advance both technical and managerial safeguards. Technically, image anonymization, sensitive-area blurring, and target segmentation can reduce the collection and exposure of non-target information. Managerially, clear data-use boundaries, storage requirements, access permissions, and result-disclosure procedures should be established to ensure that UAV thermal imaging can be used efficiently and compliantly in engineering practice.

A limitation of this study is the absence of systematic ground-truth validation through inspection (e.g., field opening, core sampling, or moisture content testing). The diagnostic results presented in this study are based on thermal anomaly patterns, temporal consistency, and spatial logic, rather than direct physical confirmation of the underlying wall construction. As such, the reliability improvement in this study refers to the reduction in temporal false positives (i.e., anomalies that appear in single-moment observations but do not persist across multiple time periods), not to the confirmation of internal structural defects. Future work will incorporate field-opening verification for a subset of high-priority anomalies to establish precision/recall metrics against ground-truth defect conditions.

In addition, summer daytime thermal imaging does not directly measure winter heat-loss rates. The anomalies identified in this study should be interpreted as indicators of potential construction weaknesses (e.g., thermal inertia variations, construction discontinuities, or material degradation) rather than direct quantification of envelope heat loss. For retrofit planning, these indicators should be combined with winter-season IRT surveys, on-site inspection, and building energy modeling to establish actual heat-loss priorities. Future work will include winter heating-season acquisitions to directly compare summer-identified anomalies with winter heat-loss patterns.

## 5. Conclusions

This study proposes an integrated method for pre-retrofit diagnosis of facade thermal defects in old residential buildings by combining multi-temporal UAV thermal imaging, 3D path planning, thermal anomaly recognition, facade spatial mapping, and temporal stability screening. Based on typical residential community cases in Wuhan’s hot-summer and cold-winter climate zone, the following conclusions are drawn.

(1) UAV thermal imaging data quality is significantly affected by sensor preheating, imaging distance, and acquisition timing. The experiments show that preheating for no less than 10 min before acquisition can reduce the influence of thermal drift in uncooled infrared detectors; controlling the UAV-to-facade distance within 8–10 m balances measurement stability, facade coverage, spatial resolution, and flight safety; and acquisition around 17:00 in summer can better reveal structural heat storage and stable thermal anomalies.
(2) (3D-model-assisted path planning improves the continuity and comparability of thermal imaging for complex residential facades. Through target-facade extraction, candidate viewpoint generation, occlusion checking, layered scanning, and U-shaped serpentine route organization, the method reduces uneven coverage and scale variation commonly found in traditional two-dimensional flight routes and provides a stable data basis for anomaly localization.
(3) The image-recognition workflow combining adaptive thresholding, Canny edge extraction, edge traversing, and region growth can extract thermal anomaly candidates from UAV thermal images. After mapping the candidates to facade views derived from 3D models, anomalies can be localized to specific floors, bays, and construction nodes, improving the engineering readability of thermal imaging results.
(4) Multi-temporal screening effectively reduces false positives caused by transient disturbances. Repeated weekday/weekend and morning–noon–afternoon observations show that some anomalies are highly time-dependent and are related to occupant behavior, air-conditioner exhaust, shading, and solar radiation, whereas recurrent anomalies around window–wall joints, floor-slab lines, wall patches, and moisture-crack areas show stronger constructional relevance and are more suitable for field verification and retrofit-priority assessment.
(5) The case study demonstrates that the method can generate diagnostic outputs from thermal acquisition and anomaly recognition to spatial expression and retrofit-oriented translation. The results from Chagang Community, Engineering West Area, and Engineering Building 65 show that different building types and retrofit statuses correspond to different anomaly distributions. Renovated buildings may still exhibit node-level and local patch-like anomalies; unrenovated buildings are more likely to exhibit large wall-surface anomalies; and high-rise buildings show more concentrated recurrent anomalies around window–wall joints and slab lines.

Overall, this study responds to common problems in conventional building thermography, including measurement error, weak temporal comparability, discontinuous spatial coverage, and difficulty in connecting diagnostic results with retrofit decisions. It provides a practical UAV-IRT workflow for thermal defect detection and energy-efficiency retrofitting of old residential communities. Future work should expand sample size, incorporate visible-light crack recognition, LiDAR point clouds, BIM models, and field-opening results, and establish more precise defect-classification models and retrofit-effect evaluation methods.

## Figures and Tables

**Table 1:** Main parameters of the UAV thermal imaging camera.

Parameter	Value
Resolution	640 × 512
Frame Rate	30 Hz
Field of View	61°
Equivalent Focal Length	40 mm
Temperature Accuracy	±2 °C
Noise Equivalent Temperature Difference (NETD)	≤50 mK

**Table 2:** Control parameters for UAV thermal imaging data acquisition.

Parameter Type	Control Content	Treatment/Setting
Acquisition month	Summer high-temperature period	Field campaigns during the hot days of July–August
Acquisition time	Intra-day multi-period comparison	Three acquisitions at 09:00, 14:00, and 17:00
Context setting	Differences in residential behavior	One weekday and one weekend selected for comparison
Weather condition	Avoid rain, strong wind, and rapid cloud changes	Acquire data under relatively stable weather
Sensor preheating	Reduce thermal drift of the uncooled infrared sensor	Preheat for no less than 10 min before takeoff
Imaging distance	Control spatial resolution and measurement stability	Keep the UAV-to-facade distance within 8–10 m
Viewing angle	Reduce oblique-viewing error	Keep the camera as perpendicular to the facade as possible
Image overlap	Ensure continuous facade coverage	Use layered U-shaped serpentine paths with necessary overlap
Emissivity setting	Maintain consistent thermal measurement parameters	Keep settings uniform during acquisition and avoid random batch-level adjustment
Data display	Avoid misinterpretation caused by palette differences	Use a unified palette mode and temperature display range
Acquisition month	Summer high-temperature period	Field campaigns during the hot days of July–August
Acquisition time	Intra-day multi-period comparison	Three acquisitions at 09:00, 14:00, and 17:00

**Table 3:** Thermal imaging path planning workflow assisted by a 3D model.

Steps	Main Content	Control Objective
3D model construction	Generate a 3D model of the building and surrounding environment from visible-light images	Identify building height, massing, occlusions, and safety boundaries
Target facade extraction	Define the facade area, floor height, and key construction details to be inspected	Determine the thermal imaging target
Candidate viewpoint generation	Generate candidate shooting points according to the 8–10 m distance, camera FOV, and facade height	Maintain consistent imaging distance and scale
Occlusion and safety check	Remove points affected by trees, pipelines, adjacent buildings, and protruding balconies	Ensure flight safety and image usability
Route sequencing	Organize a U-shaped serpentine path according to layered scanning, horizontal advancement, and vertical transition	Ensure continuous facade coverage
Supplementary imaging	Set additional viewpoints for low-floor occlusions, corners, and blind areas	Reduce data omission
Field adjustment	Slightly adjust the route according to wind, pedestrian activity, and temporary obstacles	Improve operational feasibility
Step	Main content	Control objective
3D model construction	Generate a 3D model of the building and surrounding environment from visible-light images	Identify building height, massing, occlusions, and safety boundaries
Target facade extraction	Define the facade area, floor height, and key construction details to be inspected	Determine the thermal imaging target
Candidate viewpoint generation	Generate candidate shooting points according to the 8–10 m distance, camera FOV, and facade height	Maintain consistent imaging distance and scale
Occlusion and safety check	Remove points affected by trees, pipelines, adjacent buildings, and protruding balconies	Ensure flight safety and image usability

**Table 4:** Thermal anomaly classification and annotation criteria.

Thermal Anomaly Type	Visual Characteristics	Classification Basis	Annotation Method
Thermal leakage	Relatively uniform heat loss, stable temperature distribution, and clear boundary	Obvious heat leakage, often at joints or poorly sealed envelope parts	Annotation boxes should tightly cover the region with uniform temperature deviation and clear contrast
Thermal bridge	Local temperature rise, commonly at structural joints	Abnormal heat conduction at structural components such as window or door frames	Annotations are usually small and concentrated, with clear boundaries at construction joints
Structural crack	Large local temperature difference and uneven temperature variation	Heat leakage around cracks or joints, often related to settlement or material aging	Annotations should tightly cover the region with abrupt temperature change around the crack
Uneven thermal distribution	Irregular hot or cold regions with unstable distribution	Material heterogeneity, poor construction, moisture, or aging causing irregular heat distribution	Identify irregular fluctuating regions and avoid forcing them into regular linear or point patterns

**Table 5:** Comparison of thermal anomalies detected at different times and dates.

Time	Number and Main Pattern of Thermal Anomalies	Main Interference Factor	South Facade	North Facade
21 July 2025, 09:00	9 regions, mainly around windows and wall moisture areas	Occupant behavior (window opening)	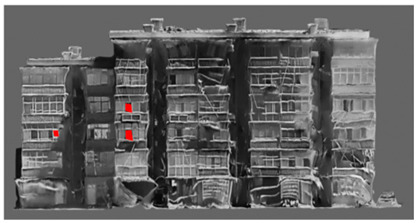	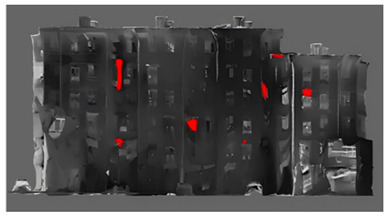
21 July 2025, 14:00	32 regions, mostly around windows, with a few wall moisture patches	Occupant behavior (window opening)	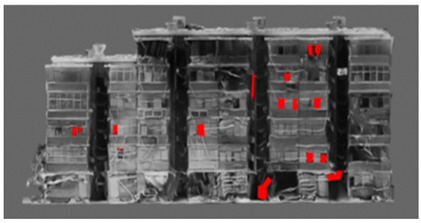	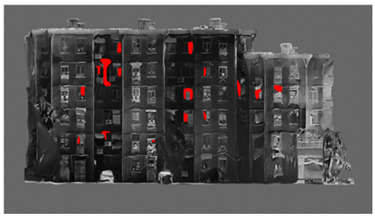
21 July 2025, 17:00	21 regions, mainly at wall corners and band-like moisture areas	Building occlusion (temporary/additional roof)	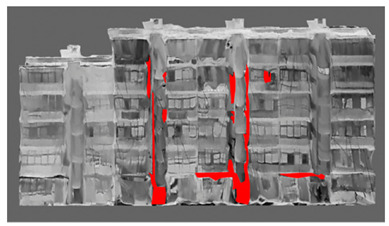	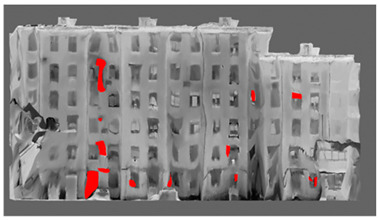
26 July 2025, 09:00	13 regions, partly around windows and wall insulation areas	Occupant behavior (window opening)	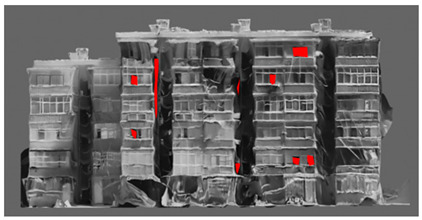	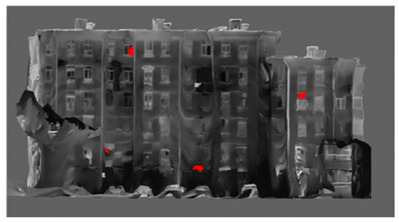
26 July 2025, 14:00	34 regions, mostly around windows, with other wall insulation-related areas	Occupant behavior (window opening)	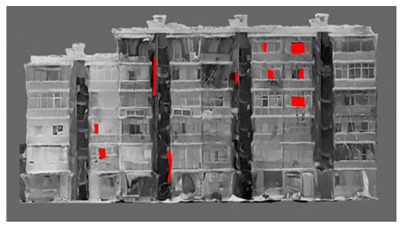	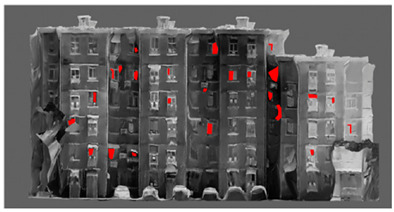
26 July 2025, 17:00	40 regions, partly around windows and partly patch-like wall insulation areas	Occupant behavior (window opening) and constructional factors	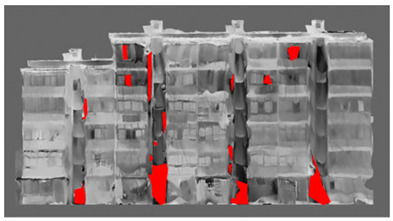	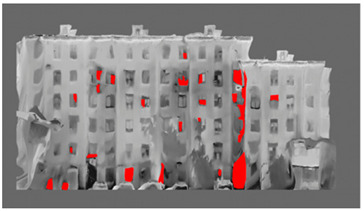

**Table 6:** Spatial localization results of thermal anomalies after temporal screening.

Building	Facade	No. of Thermal Anomalies	Diagnostic Image A	Diagnostic Image B
Chagang Community 101	A: North facade; B: South facade	26	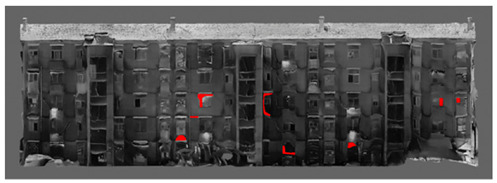	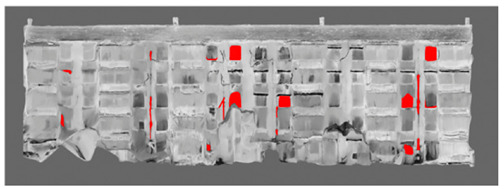
Chagang Community 102	A: North facade; B: South facade	15	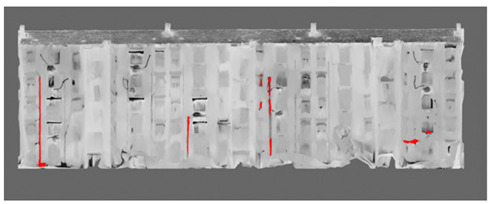	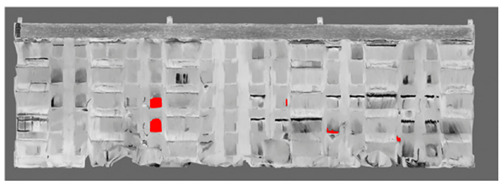
Chagang Community 103	A: North facade; B: South facade	14	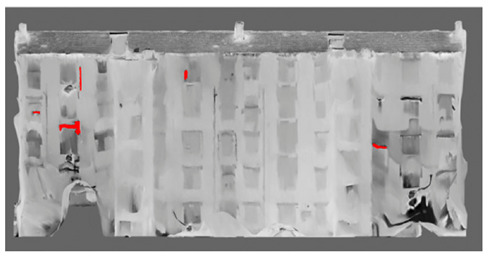	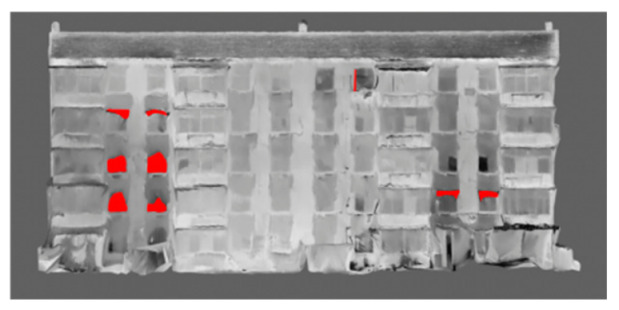
Chagang Community 104	A: North facade; B: South facade	38	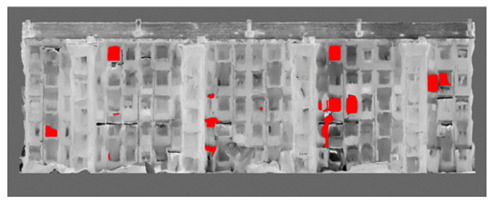	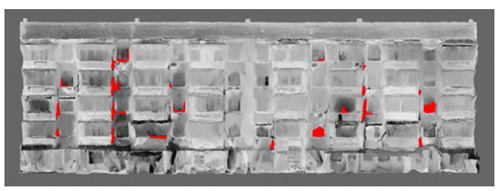
Chagang Community 105	A: North facade; B: South facade	29	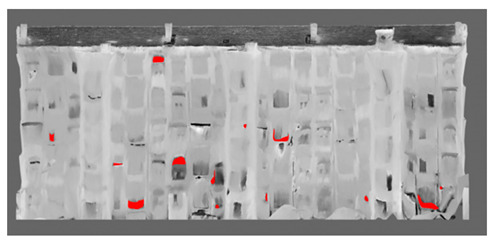	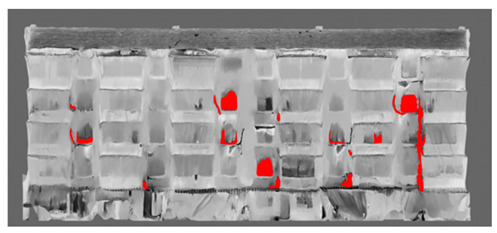
Chagang Community 106	A: North facade; B: South facade	24	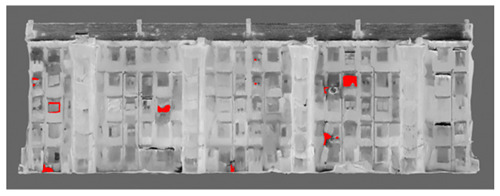	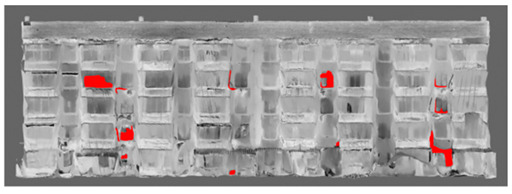
Engineering West Area 59	A: North facade; B: South facade	19	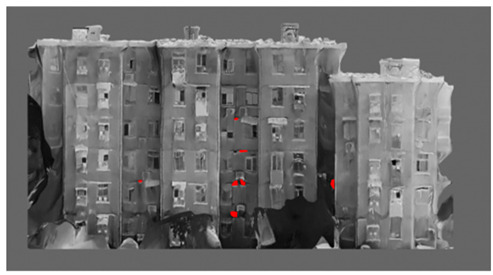	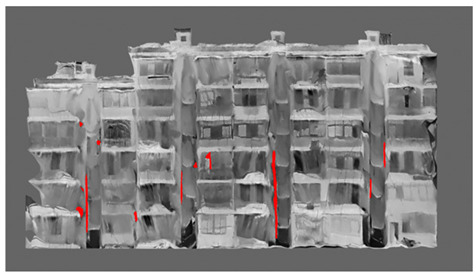
Engineering West Area 60	A: North facade; B: South facade	16	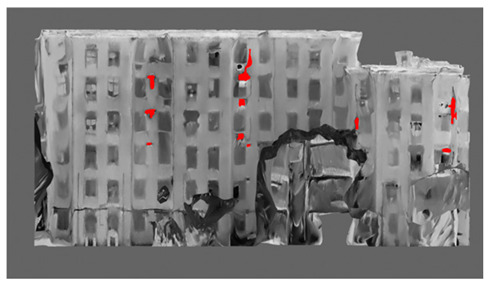	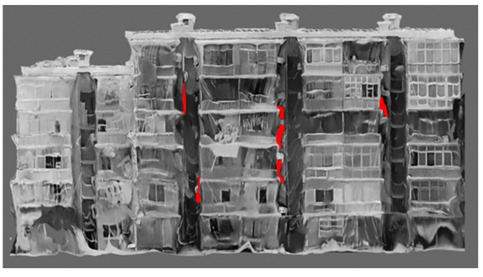
Engineering West Area 61	A: North facade; B: South facade	26	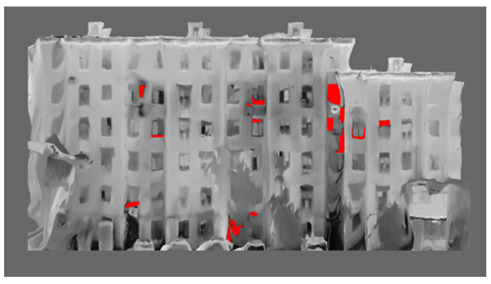	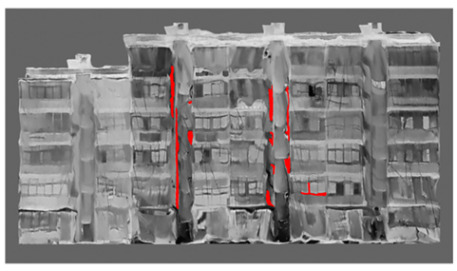
Engineering West Area 62	A: North facade; B: South facade	40	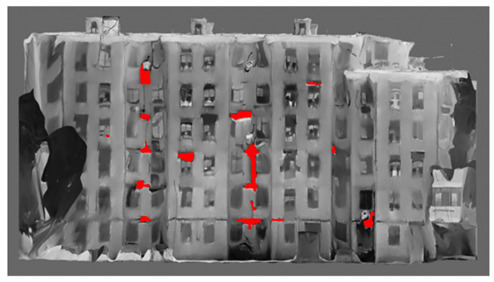	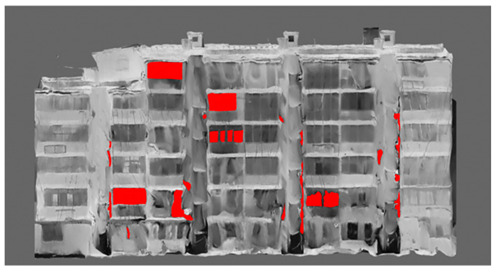
Engineering Building 65	A: North facade; B: South facade	69	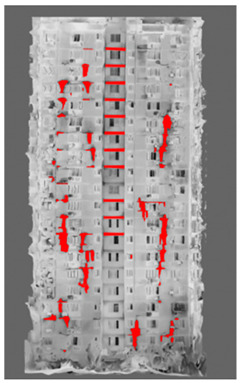	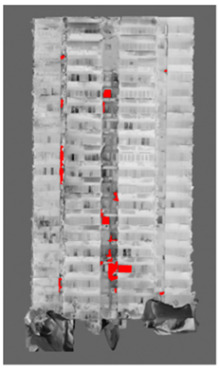
Engineering Building 65	A: East facade; B: West facade	43	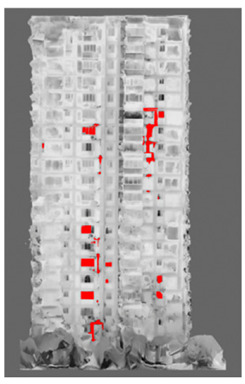	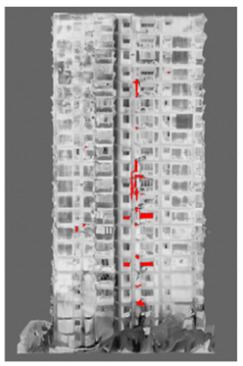

## Data Availability

The data presented in this study are available on request from the corresponding author.

## References

[1] Hwaish A.N.A. (2015). Impact of heat exchange on building envelope in the hot climates. Int. J. Emerg. Technol. Adv. Eng..

[2] Ministry of Housing and Urban-Rural Development of China Notification on Issuing the 14th Five-Year Plan for Building Energy Efficiency and Green Building Development. https://www.mohurd.gov.cn/gongkai/zc/wjk/art/2022/art_17339_765109.html.

[3] Wuhan Municipal People’s Government Wuhan Green Building Management Measures. https://www.wuhan.gov.cn/whszfwz/wjk/gz/202309/t20230914_2264452.shtml.

[4] Garrido I., Lagüela S., Otero R., Arias P. (2020). Thermographic methodologies used in infrastructure inspection: A review—Post-processing procedures. Appl. Energy.

[5] Lucchi E. (2018). Applications of the infrared thermography in the energy audit of buildings: A review. Renew. Sustain. Energy Rev..

[6] Tomita K., Chew M.Y. (2022). A Review of Infrared Thermography for Delamination Detection on Infrastructures and Buildings. Sensors.

[7] Jiang T., Hao F., Chen X., Zou Z., Zheng S., Liu Y., Xu S., Yin H., Yang X. (2024). Estimating indoor air temperature by obtaining outdoor building window surface temperature using infrared technology: An exploratory approach. Build. Environ..

[8] Li Z., Jin Y., Liang X., Zeng J. (2022). Thermography evaluation of defect characteristics of building envelopes in urban villages in Guangzhou, China. Constr. Mater..

[9] Baldinelli G., Bianchi F., Rotili A., Costarelli D., Seracini M., Vinti G., Asdrubali F., Evangelisti L. (2018). A model for the improvement of thermal bridges quantitative assessment by infrared thermography. Appl. Energy.

[10] Tabet Aoul K.A., Hagi R., Abdelghani R., Syam M., Akhozheya B. (2021). Building Envelope Thermal Defects in Existing and Under-Construction Housing in the UAE; Infrared Thermography Diagnosis and Qualitative Impacts Analysis. Sustainability.

[11] Fokaides P.A., Kalogirou S.A. (2011). Application of infrared thermography for the determination of the overall heat transfer coefficient (U-Value) in building envelopes. Appl. Energy.

[12] Martínez I., Martínez E. (2022). Qualitative timber structure assessment with passive IR thermography. Case study of sources of common errors. Constr. Mater..

[13] Edis E., Flores-Colen I., de Brito J. (2014). Passive thermographic detection of moisture problems in façades with adhered ceramic cladding. Constr. Build. Mater..

[14] Lu X., Memari A. (2019). Application of infrared thermography for in-situ determination of building envelope thermal properties. J. Build. Eng..

[15] Fox M., Goodhew S., De Wilde P. (2016). Building defect detection: External versus internal thermography. Build. Environ..

[16] Mayer Z., Heuer J., Volk R., Schultmann F. (2021). Aerial Thermographic Image-Based Assessment of Thermal Bridges Using Representative Classifications and Calculations. Energies.

[17] Zhang D., Zhan C., Chen L., Wang Y., Li G. (2024). An in-situ detection method for assessing the thermal transmittance of building exterior walls using unmanned aerial vehicle–infrared thermography (UAV-IRT). J. Build. Eng..

[18] Zhu L., Hurt R., Correia D., Boehm R. (2009). Detailed energy saving performance analyses on thermal mass walls demonstrated in a zero energy house. Energy Build..

[19] Benhmidou H., Romani Z., El Mankibi M., Draoui A. (2021). Thermal performance prediction of an existing building with framing system using the IRT method. Adv. Build. Energy.

[20] Mohammad S., Shea A. (2013). Performance Evaluation of Modern Building Thermal Envelope Designs in the Semi-Arid Continental Climate of Tehran. Buildings.

[21] Sadeghifam A.N., Marsono A.K., Kiani I., Isikdag U., Bavafa A.A., Tabatabaee S. (2016). Energy analysis of wall materials using building information modeling (BIM) of public buildings in the tropical climate countries. J. Teknol..

[22] Kisilewicz T. (2019). On the Role of External Walls in the Reduction of Energy Demand and the Mitigation of Human Thermal Discomfort. Sustainability.

[23] de Freitas S.S., de Freitas V.P., Barreira E. (2014). Detection of façade plaster detachments using infrared thermography–A nondestructive technique. Constr. Build. Mater..

[24] Zhang D., Zhan C., Chen L., Wang Y., Li G. (2025). Review of unmanned aerial vehicle infrared thermography (UAV-IRT) applications in building thermal performance: Towards the thermal performance evaluation of building envelope. Quant. InfraRed Thermogr. J..

[25] Mirzabeigi S., Razkenari R., Crovella P. (2025). A Review of the Potential of Drone-Based Approaches for Integrated Building Envelope Assessment. Buildings.

[26] Kylili A., Fokaides P.A., Christou P., Kalogirou S.A. (2014). Infrared thermography (IRT) applications for building diagnostics: A review. Appl. Energy.

[27] Bayomi N., Nagpal S., Rakha T., Fernandez J.E. (2021). Building envelope modeling calibration using aerial thermography. Energy Build..

[28] Nagasawa R., Mas E., Moya L., Koshimura S. (2021). Model-based analysis of multi-UAV path planning for surveying postdisaster building damage. Sci. Rep..

[29] Entrop A.G., Vasenev A. (2017). Infrared drones in the construction industry: Designing a protocol for building thermography procedures. Energy Procedia.

[30] Ellefsen K.O., Lepikson H.A., Albiez J.C. (2017). Multiobjective coverage path planning: Enabling automated inspection of complex, real-world structures. Appl. Soft Comput..

[31] Huang X., Liu Y., Huang L., Stikbakke S., Onstein E. (2023). BIM-supported drone path planning for building exterior surface inspection. Comput. Ind..

[32] Jung S., Song S., Youn P., Myung H. Multi-Layer Coverage Path Planner for Autonomous Structural Inspection of High-Rise Structures. Proceedings of the 2018 IEEE/RSJ International Conference on Intelligent Robots and Systems (IROS).

[33] Bauer E., de Freitas V.P., Mustelier N., Barreira E., de Freitas S.S. (2015). Infrared thermography–evaluation of the results reproducibility. Struct. Surv..

[34] Vollmer M., Möllmann K.-P. (2018). Infrared Thermal Imaging: Fundamentals, Research and Applications.

[35] Bircher A., Kamel M., Alexis K., Burri M., Oettershagen P., Omari S., Mantel T., Siegwart R. (2016). Three-dimensional coverage path planning via viewpoint resampling and tour optimization for aerial robots. Auton. Robots.

[36] Biundini I.Z., Pinto M.F., Melo A.G., Marcato A.L.M., Honório L.M., Aguiar M.J.R. (2021). A Framework for Coverage Path Planning Optimization Based on Point Cloud for Structural Inspection. Sensors.

[37] Ibarra-Castanedo C., Sfarra S., Klein M., Maldague X. (2017). Solar loading thermography: Time-lapsed thermographic survey and advanced thermographic signal processing for the inspection of civil engineering and cultural heritage structures. Infrared Phys. Technol..

[38] Canny J. (1986). A computational approach to edge detection. IEEE Transactions on Pattern Analysis Machine Intelligence.

[39] Adams R., Bischof L. (1994). Seeded region growing. IEEE Transactions on Pattern Analysis Machine Intelligence.

